# Neuroprotective effects of candesartan in 3-nitropropionic acid-induced Huntington’s disease: modulation of angiotensin and CREB/BDNF/PGC1-α signaling

**DOI:** 10.1007/s10787-025-01889-6

**Published:** 2025-09-16

**Authors:** Ali M. Elgindy, Ahmed M. Atwa, El-Sayed E. El-Awady, Norhan M. El-Sayed, Naglaa F. El-Orabi

**Affiliations:** 1 Department of Pharmacology and Toxicology, Faculty of Pharmacy, Egyptian Russian University, Cairo, 11829 Egypt; 2 College of Pharmacy, Al-Ayen Iraqi University, AUIQ, An Nasiriyah, Iraq; 3 Department of Pharmacology and Toxicology, Faculty of Pharmacy, Suez Canal University, Ismailia, 41522 Egypt

**Keywords:** Huntington’s disease, Candesartan, Neuroprotection, Renin–Angiotensin System, CREB/BDNF/PGC1-α signaling, JNK/c-Jun pathway

## Abstract

Renin–Angiotensin System has been implicated in neurodegenerative diseases such as Huntington’s (HD). It is made up of two axes: one is the Angiotensin II Type 1 receptor (AT1R) or Angiotensin II Type 2 receptor (AT2R), while the other is angiotensin-(1-7) [Ang-(1-7)], and Mas receptor; the latter has reported developing a neuroprotective effect; oppositely, AT1R activation has been linked to neurodegenerative diseases. This study aims to elucidate the potential neuroprotective effect of Candesartan (Cand) an AT1R blocker in mitigating neuronal degeneration caused by 3-Nitropropionic acid (3NP) induced HD by revealing the prospective role of Ang II/AT2R/Ang-(1-7)/Mas receptor, CREB/BDNF/PGC1-α besides JNK/c-Jun trajectories, as well as the anti-apoptotic survivin. HD was induced by 3NP (10 mg/kg) for 14 days. Rats received Candesartan (2.5 or 5 mg/kg) for 14 days, after which brain and striatum were isolated for the histopathological, immunohistochemical, and biochemical analysis. Cand displayed significant improvement in the rats’ behavioral tests, enhancing their memory, motor, and cognitive functions induced by 3NP, which was confirmed by the striatal histopathological and immunohistochemical examination of the GFAP. In addition, Cand activated the Ang II/AT2R/Ang-(1-7)/Mas receptor axis. Moreover, Cand stimulated the production of striatal neurotrophic proteins CREB/BDNF/PGC1-α which in turn decreased the levels of inflammatory mediators NF-κB and IL-1β, accompanied by an increase in the antioxidants NQO1 and HO-1 levels. Similarly, Cand led to the inhibition of the JNK/c-Jun with activation of survivin. In conclusion, Candesartan has mitigated the striatal degeneration and mitochondrial dysfunction induced by 3NP through various mechanistic pathways.

**Supplementary Information:**

The online version contains supplementary material available at 10.1007/s10787-025-01889-6.

## Introduction

Huntington’s disease (HD) is a progressive neurodegenerative disorder primarily affecting the basal ganglia. It is characterized by motor dysfunction (notably involuntary choreiform movements), cognitive decline, and psychiatric disturbances (Vinther-Jensen et al. 2014). The pathogenesis of HD involves mitochondrial dysfunction, impaired energy metabolism, oxidative stress, excitotoxicity, transcriptional dysregulation, and disrupted synaptic transmission (Ferguson et al. 2022a). Although the exact mechanisms remain incompletely understood, HD is caused by an unstable expansion of a CAG trinucleotide repeat in the huntingtin (HTT**)** gene, leading to the production of mutant huntingtin protein. This mutant protein aggregates predominantly in the striatum, particularly in the putamen and caudate nucleus, causing progressive neuronal loss (Ramaswamy et al. 2007; Ross and Tabrizi 2011; McColgan and Tabrizi 2018).

The expression of the mutant huntingtin gene is widely recognized in the cardiomyocytes of Huntington patients, resulting in cardiac failure and hypertension, which is the primary reason for death in those patients. Meanwhile, cardiovascular diseases (CVS) such as cardiac failure and hypertension (HTN) can negatively impact the function and the structure of the brain, hastening the progression of neurodegenerative diseases such as HD (Burg et al. 2009; Steventon et al. 2020; Schultz et al. 2020). In this context, the Renin–Angiotensin System (RAS) which exists in the central and peripheral nervous system has been implicated in neuropsychiatric and neurodegenerative diseases as HD besides CVS diseases as HTN and cardiac failure (Mello et al. 2017a; Pugliese et al. 2020; Machado et al. 2020; Villapol 2023; Ahmadi and Khaledi 2023).

The RAS consists of two primary axes: the first axis is composed of angiotensin-converting enzyme (ACE), which transforms angiotensin I (Ang I) into angiotensin II (Ang II). In turn, Ang II binds to the Angiotensin II Type 1 receptor (AT1R) or Angiotensin II Type 2 receptor (AT2R). Depending on the receptor that Ang II binds to, several physiological responses might be observed. Nonetheless, the AT1 receptor is the main Ang II mediator (Machado et al. 2020; Fyhrquist and Saijonmaa 2008; Miranda and Teixeira 2023). In the CNS, the ACE/Ang II/AT1 receptor axis is often linked to neurodegenerative disease, because it stimulates the accumulation of inflammatory markers and free radicals, and decreases neuronal survival (Gironacci, et al. 2018; Rocha et al. 2016; Almeida-Santos et al. 2017).

On the other hand, the components of the second axis, which is the counter-regulatory axis formed of ACE [Angiotensin-Converting Enzyme 2 (ACE2)], angiotensin-(1-7) [Ang-(1-7)], and Mas receptor (Jankowski et al. 2007; Lautner et al. 2013; Santos, et al. 2019). Ang-(1-7) can be formed directly or indirectly from Ang I by angiotensin-converting enzyme 2 (ACE2) (Jiang et al. 2014). Interestingly, the activation of the ACE2/Ang-(1-7)/Mas receptor was reported to develop a neuroprotective action. For instance, it sustained neuronal survival, reduced oxidative stress and neuroinflammation, and improved cognitive function (Gironacci, et al. 2018; Rocha et al. 2016; Jiang et al. 2016). Furthermore, AT2 receptor activation by Ang II has comparable functions to Mas receptor activation by Ang-(1-7). It opposes the major response produced by the activation of the AT1 receptor, exerting mostly neuroprotective benefits (Almeida-Santos et al. 2017; Kangussu et al. 2022). Moreover, a recent publication noted a marked reduction in the beneficial axis ACE2/Ang-(1-7)/Mas receptor in the striatum of HD brain tissues. On the contrary, the Ang II/AT1R axis was significantly activated in brain tissues (Kangussu et al. 2022).

Emerging evidence supports a pivotal role for RAS in HD. For example, human post-mortem studies have revealed reduced ACE activity in HD-affected brain regions, including the striatum and substantia nigra. Additionally, striatal ACE2 activity and the levels of Angiotensin-(1-7) are decreased in HD mouse models, while AT1 receptor expression is increased. These findings demonstrate a shift in RAS balance toward the pathological, pro-inflammatory classical axis and provide a strong biological rationale for targeting RAS modulation as a therapeutic strategy in HD (Kangussu et al. 2022).

In addition, further stimulation of the AT2 receptor leads to the phosphorylation of CREB via the activation of the PI3K/Akt axis (Guimond and Gallo-Payet 2012). Consequently, this leads to the activation of the BDNF protein downstream (Hashikawa-hobara et al. 2012; Umschweif et al. 2014; Guimond, et al. 2013; McFall et al. 2020). The CREB and BDNF proteins both play a crucial role in providing neuroprotection and cell survival against neurodegenerative diseases by enhancing cellular proliferation besides suppressing inflammatory biomarkers and apoptosis, eventually resulting in expanded cell survival (Jiang et al. 2013; Ribeiro et al. 2014; Heras-Sandoval et al. 2014; Zuo et al. 2016). Moreover, CREB can trigger the expression of PGC-1α, a transcriptional coactivator that plays a major role in brain energy homeostasis, therefore, hindering the mitochondrial dysfunction associated with neurodegenerative diseases (Singulani et al. 2020). Correspondingly, CREB could halt the transcription of the JNK/C-Jun axis, which has been implicated in chronic neurological disorders (Perrin et al. 2009; Zhang et al. 2019).

For a model of HD to be deemed reliable, it must possess neuropathological characteristics and symptomatic manifestations associated with the illness. 3-Nitropropionic acid (3NP) is an environmental toxin that mimics the pathology and motor abnormalities associated with Huntington’s disease (HD). 3NP irreversibly inhibits succinate dehydrogenase (SDH), disrupting the Krebs cycle and consequently reducing adenosine triphosphate (ATP) levels in the brain. Additionally, 3NP induces free radical production, impairs the brain’s antioxidant defense mechanisms, and promotes the release of pro-inflammatory cytokines, including TNF-α, NF-κB, and interleukin-1β (Haddadi et al. 2024; Jamwal and Kumar 2016). These factors contribute to the generation of excess free radicals, forthcoming impending neuronal damage (Upadhayay et al. 2023). Furthermore, it has been documented that striatal lesions induced by 3NP exhibit a high degree of resemblance to the neurochemical, histological, and morphological pathology observed in HD (Ramaswamy et al. 2007).

Currently, there is a continuous lack of appropriate curative therapy for Huntington’s disease (Ferguson et al. 2022b). There is a necessity for the pinpointing of novel targets to facilitate the advancement of therapeutic techniques. The possible neuroprotective impact of Candesartan, an AT1R blocker, has been postulated in the context of neural inflammation and neurological diseases. In particular, prior investigations have demonstrated a significant neuroprotective potential of Candesartan in AD and PD (Elkahloun et al. 2016; Gaur and Kumar 2011; Wu et al. 2013).

Accordingly, the following study was designed to elucidate the potential neuroprotective properties of Candesartan for the first time, in mitigating neuronal degeneration caused by 3NP induced HD through investigating the prospective role of Ang II/AT2R/Ang-(1-7)/Mas receptor, CREB/BDNF/PGC1-α besides JNK/c-Jun trajectories. In addition, the anti-apoptotic effect of Survivin was also highlighted in this study.

## Materials and methods

### Animals

Adult male albino Wistar rats (12 weeks old, weighing 180–200 g) were procured from the National Research Centre (NRC, Giza, Egypt). Before starting the experiment, the animals were acclimatized for 1 week under laboratory conditions at the animal facility of the Faculty of Pharmacy, Suez Canal University. They were housed in a controlled environment with a temperature of 25 ± 2 °C, 60% humidity, and a 12-h light/dark cycle. The rats had unrestricted access to food and water throughout the study, and all behavioral tests were performed in an acoustically isolated laboratory. Animal care and experimental procedures adhered to the guidelines established by the Animal Care and Use Committee of the Faculty of Pharmacy, Suez Canal University (Serial number of the protocol: 202302 PhDA_1_). The study is reported in accordance with the ARRIVE guidelines (Percie du Sert, et al. 2020).

### Drugs and experimental design

Candesartan and 3-nitropropionic acid obtained from (Sigma-Aldrich, USA) were freshly prepared daily by dissolving in normal saline with pH neutralized at 7.4 using NaOH. Rats were divided randomly into 5 groups (8 rats per group). Group 1: injected with normal saline (i.p) daily for 14 days. Group 2: received Candesartan (5 mg/kg, p.o.) daily for 14 days. Groups 3–5: injected with 3NP (10 mg/kg, i.p) daily for 14 days (Danduga et al. 2018). Group 3 was considered the induction group. Groups 4–5: received Candesartan (2.5 or 5 mg/kg, p.o.) 1 h after 3-NP, respectively (Thakur et al. 2015).

By the end of the experiment, the behavioral tests were carried out with a period of 2 h rest between each test. Afterward, the rats were euthanized using I.P. injection of 100 mg/kg ketamine and 10 mg/kg xylazine, and then sacrificed by cervical dislocation. The brains were removed and divided into 2 halves. The right cerebral hemisphere was harvested rapidly and fixed in 10% neutral buffer formalin, which was used for histopathological and immunohistochemical study. While striatum was dissected from the left cerebral hemisphere and preserved at −80 °C, which was used for biochemical analysis.

### Behavioral assessment

#### Novel object recognition test (NORT)

A novel object recognition test was used to assess the recognition memory. The experiment was conducted within an open-field rectangular box with dimensions of 0.5 × 0.25 × 0.5 m. During the habituation phase, the rats were allowed to freely roam the test box in the absence of any objects for 10 min per day over two consecutive days. During the training sessions, each rat was placed solely into the box, wherein two identical objects were positioned in separate corners of the box, with an approximate distance of 0.3 m between them. During the experimental session, the animals were solely reintroduced into the test box, wherein one of the previously encountered objects was substituted for a novel object. The duration of time spent examining each object was documented for 3 min utilizing an overhead camera throughout test periods. The discrimination index was calculated by determining a difference in the duration of exploration between novel and familiar objects, divided by the overall duration of exploration for both objects. Additionally, the time allocated to examining familiar and novel things was also assessed (Agrawal et al. 2020).

#### Open-field test (OFT)

An open-field test was conducted to assess the locomotor behavior of rats. The apparatus utilized in the experiment was a wooden square box of 0.8 × 0.8 × 0.4 m. The box encompassed red walls and a black polished floor, which was subdivided by white lines into 16 squares of equal size. Each rat was solely positioned in the central location of the device and given 5 min to explore the field. The animals were monitored using an above camera to observe their ambulation frequency, which defines the square number each rat has crossed, and their rearing frequency, which defines the number of stretches on the rat’s hind limbs. Following the completion of each animal’s testing, the floor underwent a thorough cleaning process (Ramachandran and Thangarajan 2018).

#### Morris water maze (MWM)

Morris water maze test was used to evaluate spatial learning and memory retention in rats. The animals underwent training to navigate toward a platform located within a circular pool. The pool had a diameter of 1.5 m and a height of 0.6 m, with inner surfaces that were non-reflective. The pool was divided into four quadrants and filled with water up to a level of 0.35 m. The water in the pool was maintained at a constant temperature of 25 ± 2 °C. For the acquisition test, a circular platform with a diameter of 9 cm was positioned in the center of a designated quadrant within the pool. The platform was placed 1 cm below the surface of the water. To render the water opaque, a soluble black paint that is non-toxic was employed. In the initial phase of the experiment, the rats had a total of three training sessions per day, each lasting 120 s, over four consecutive days starting on the eleventh day. During these sessions, the rats were allowed to freely navigate and locate the platform from various starting points. If the rat failed to locate the designated platform, it was subsequently directed toward it and allowed to remain on it for 30 s. On the fifteenth day of the experiment, a probe test was conducted. During this test, the platform was removed, and the animal was released into the pool, facing the wall in the quadrant opposite to the target quadrant. The animal was given 1 min to explore the pool. The duration of the animal’s swimming activity in the designated quadrant was documented by the utilization of an overhead camera (Suganya and Sumathi 2017).

### Histopathological examination

Brain specimens from rats in various experimental groups were collected and preserved in a 10% neutral buffer formalin solution. Subsequently, the specimens were trimmed, rinsed with water, dehydrated using ascending grades of ethyl alcohol, clarified with xylene, and finally embedded in paraffin. A thin section with a thickness ranging from 4 to 6 µm was prepared and subjected to staining using the Hematoxylin & Eosin stain (Bancroft and Gamble 2008). To quantitatively assess histopathological alterations, perineuronal edema and astrogliosis were semi-quantitatively scored using ImageJ software. Each field was scored manually on a severity scale from 0 (normal) to 20 (severe).

### Immunohistochemical examination of glial fibrillary acidic protein (GFAP)

Striatal paraffin sections were deparaffinized and washed with PBS for 10 min, followed by incubation in 3% hydrogen peroxide for another 10 min. The specimens were then rinsed with distilled water and mounted on positively charged slides using the avidin–biotin–peroxidase complex (ABC) method. Sections were incubated with a mouse GFAP monoclonal antibody (Servicebio, Cat. No. GB12090-100, Dilution: 1:500). Subsequently, reagents for the ABC method, including the Vectastain ABC-HRP kit (Vector Laboratories), were applied. Antigen-antibody binding was identified using peroxidase labeling and visualized with diaminobenzidine (DAB) (Sigma-Aldrich, USA). Negative controls were included by replacing either the primary or secondary antibodies with non-immune serum. The immunohistochemically stained sections were examined under an Olympus BX-53 microscope, and the GFAP area percentage in the striatum was quantified using ImageJ software version 1.4 (NIH, LOCI, United States).

### Assessment of parameters

#### Body weight

The measurement of body weight was conducted at the beginning and end of the experiment. The percentage change in weight was then determined by comparing the final measurement with the starting measurement taken on the first day. Nevertheless, there was no mortality found with the injection of 3NP.

#### Western blot analysis of phospho-CREB (Ser133), anti-angiotensin-(1-7) Mas receptor, and anti-type-2 angiotensin II receptor

Striatal protein extraction was done using radioimmunoprecipitation assay (RIPA) buffer (Bio Basic Inc, Ontario, Canada). The lysate product was incubated on ice for 30 min; furthermore, centrifugation for 30 min at 16,000×g at 4 ºC was done for the removal of cell debris. The supernatant was separated into a new tube, and for determination of protein concentration, Bradford Protein Assay Kit (Bio Basic Inc, Ontario, Canada) was used. 20 µg of protein concentration was added to Laemmli buffer and boiled for 5 min, and then separated using SDS-PAGE, which was later transferred to PVDF membrane. To prevent nonspecific binding of the antibodies to the membrane, Tris-buffered saline with Tween 20 (TBST) buffer in addition to bovine serum albumin 3% (BSA) was used to block the membrane for 1 h at room temperature. Protein expression was assessed by subjecting the membrane to incubation with primary antibodies against Phospho-CREB (Ser133) Polyclonal Antibody (1:200, Cat no. PA5-97331, Thermo Fisher Scientific, United States**),** Anti-Angiotensin-(1-7) Mas Receptor Antibody (1:200, Cat no. AAR-013 Alomone Labs, United States), Anti-Angiotensin II Receptor Type-2 Antibody (1:400, Cat no. AAR-012 Alomone Labs, United States), and beta-Actin Polyclonal Antibody (1:1000, Cat no. PA5-16914 Thermo Fisher Scientific, United States) at 4 ºC overnight. Afterward, the Goat anti-Rabbit IgG (1:10000, Cat no. A16110 Thermo Fisher Scientific, United States) secondary antibody was added and incubated at room temperature for 1 h. Densitometric analysis was used to quantify the targeted protein using the ChemiDoc™ MP Imaging System. Beta-actin protein expression was used for normalization of the results, which were expressed as arbitrary units (AU).

#### ELISA Assay of Nuclear Factor-kappa B p65 (NF-κB p65), Interleukin-1 beta (IL-1β), Heme Oxygenase 1 (HO-1), NAD(P)H Quinone Dehydrogenase 1 (NQO1), and Peroxisome proliferator-activated receptor gamma coactivator 1 alpha (PGC-1α)

ELISA assay was used to quantify the protein levels of Nuclear Factor-kappa B p65 (NF-κB p65), Interleukin-1 beta (IL-1β), Heme Oxygenase 1 (HO-1), NAD(P)H Quinone Dehydrogenase 1 (NQO1), and Peroxisome proliferator-activated receptor gamma coactivator 1 alpha (PGC-1α) in the striatum. ELISA kits were procured from MyBiosource, Inc (CA, USA), and protein levels were measured according to the manufacturer’s procedures (NF-κB, Cat no. MBS2505513), (IL-1β, Cat no. MBS825017), (HO-1, Cat no. MBS764989), (NQO1, Cat no. MBS7606601), and (PGC-1α, Cat no. MBS1600213). The result values related to the NF-κB p65, IL-1β, and NQO1 were presented as pg/mg protein, while for HO-1 and PGC-1α, the result values were presented as ng/mg protein.

#### PCR assay of c-Jun N-terminal kinase (JNK), c-Jun, Survivin, brain-derived neurotrophic factor (BDNF) and Caspase-3

For the determination of the protein expression of c-Jun N-terminal kinase (JNK), c-Jun, Survivin, brain-derived neurotrophic factor (BDNF) and Caspase-3, the tissue homogenate was processed for RNA extraction followed by reverse transcriptase (for cDNA synthesis) and quantitative real-time PCR. For the extraction of the total RNA from striatal homogenate, the SV total RNA isolation system (Thermo Scientific, USA) was used, and the yield of the obtained total RNA was measured using a spectrophotometer at 260 nm. For the cDNA synthesis, 1μg of the total RNA was used with the cDNA reverse transcription kit (K4374966, Thermo Fisher Scientific, USA). Quantification using RT-PCR was executed on JNK, c-Jun, Survivin, BDNF, and caspase-3 according to the SYBR Green Master Mix (Applied Biosystems, CA, USA). The qPCR assay with the primer sets is displayed in (Table 1). Finally, principles and procedures for the preparation of the reaction master mix for RT-PCR were done according to a previous study (Afonina et al. 1997). The running condition was 50 °C for 2 min, 95 °C for 10 min, and 40 cycles of 95 °C for 15 s and 60 °C for 60 s. The results were measured using the cycle threshold (Ct) method. The results were displayed as relative fold changes in comparison to the expression of the control gene (GAPDH).

**Table 1:** Primers used in RT-qPCR

Gene	Accession number	Forward (5′–3′)	Reverse (5′–3′)	GC content (%)
JNK	XM_054366304.1	ATCCAGCAGAAGCAAGCG	GCCAGACCGAAGTCAAGAATC	55.5 52.3
cJun	NM_002228.4	GCCTACAGATGAACTCTTTCTGGC	CCTGAAACATCGCACTATCCTTTG	50.0 45.8
Survivin	NM_001012270.2	TCCACTGCCCCACTGAGAAC	TGGCTCCCAGCCTTCCA	60.0 64.7
BDNF	NM_170734.4	CGAAGAGCTGCTGGATGAG	ATGGGATTACACTTGGTCTCG	57.9 47.6
Caspase-3	XM_054350956.1	CGAAACTCTTCATCATTCAGGC	AGTAAGCATACAGGAAGTCGGC	45.45 50.00
GAPDH	NM_001357943.2	GTCTCCTCTGACTTCAACAGCG	ACCACCCTGTTGCTGTAGCCAA	54.5 54.5

JNK: c-Jun N-terminal kinase, BDNF: brain-derived neurotrophic factor, GAPDH: Glyceraldehyde 3-phosphate dehydrogenase

### Statistical analysis

The data obtained were presented as the mean ± SD. For all parameters, comparing among groups was analyzed using a one-way analysis of variance test (one-way ANOVA) followed by Tukey’s test. However, the Kruskal–Wallis test followed by Dunn’s test was used to analyze the ambulation and rearing frequency results obtained from the open-field test and histological score of perineuronal edema and astrogliosis. Significance testing for differences in exploration duration between familiar and novel objects was conducted for each group using a two-way analysis of variance (ANOVA), with objects and drug treatments as fixed factors. All the statistical analyses were carried out using GraphPad Prism (version 8.0.2). For all tests, the results were considered significant at *p* < 0.05.

## Results

### Effect of candesartan on body weight of rats in 3np induction model

The injection of 3NP (10 mg/kg) over 14 days resulted in noticeable changes in the animals’ body weight (Table 2). Specifically, the 3NP group showed a significant 17.53% reduction in final body weight compared to the normal group. Co-treatment with Candesartan at doses of 2.5 mg/kg and 5 mg/kg for 14 days significantly raised body weight by 1.07-fold and 1.1-fold, respectively, relative to the 3NP group. However, no statistically significant difference was observed between the two Candesartan-treated groups.

**Table 2:** Effect of Candesartan on the changes in body weight of 3NP induced rats

Group	At the beginning of the model	At the end of the model	Percentage change in body weight (%)
Control	200.55 ± 7.38	209.57 ± 7.71	4.49
Cand 5	201.98 ± 12.08	208.96 ± 12.49	3.45
3NP	188.33 ± 5.92	172.82 ± 4.84*	-8.23
3NP + Cand 2.5	182.2 ± 6.09	185.85 ± 5.88*^,@^	2
3NP + Cand 5	185.55 ± 7.49	191.07 ± 8.45*^,@^	2.97

Data are represented as means ± standard deviation at *p* < 0.05 using one-way ANOVA, followed by Tukey's post hoc test

3NP, 3-nitropropionic acid; Cand, Candesartan

* Versus control, @ versus 3NP

### Effect of Candesartan on the novel object recognition test in 3NP induction model

**Fig. 1:**
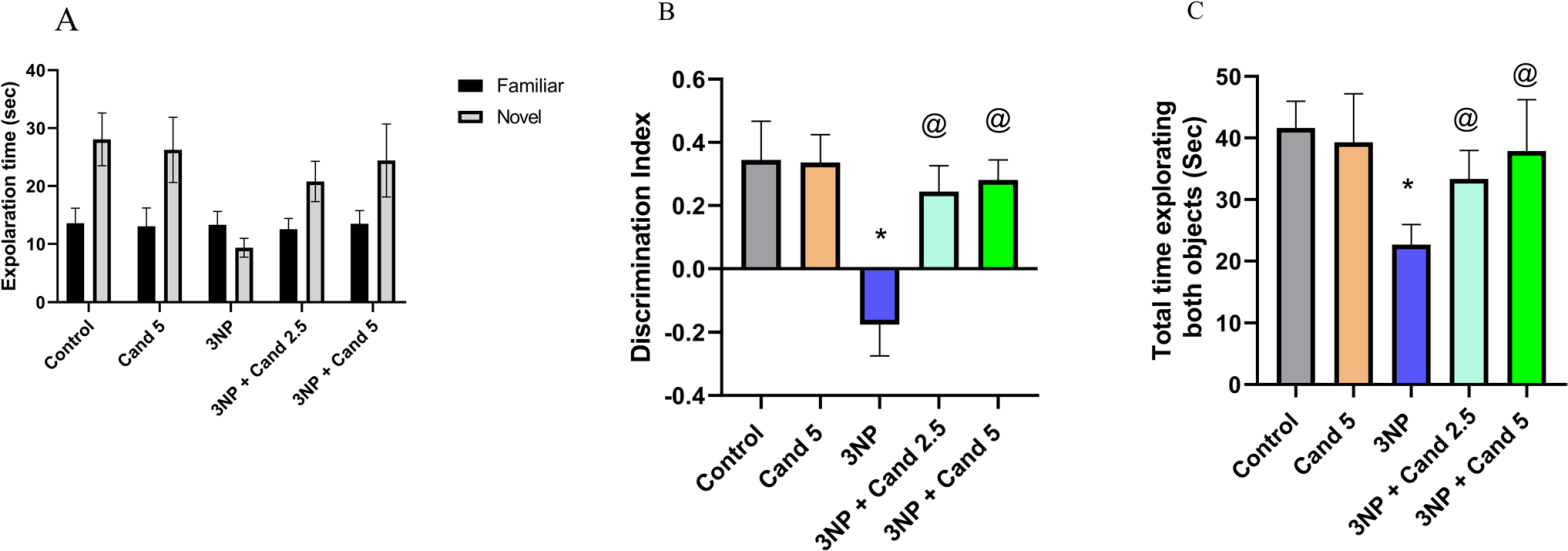
Effect of Candesartan on 3NP induced alteration in the novel object recognition test: **A** exploration time of familiar and novel objects, **B** discrimination Index, and **C** total time exploring both objects. The parametric data were provided as the mean ± standard deviation at *p* < 0.05 using one-way ANOVA, followed by Tukey’s post hoc test. Comparison between the exploration time of the familiar and novel object is measured using two-way ANOVA. * Versus control, @ versus 3NP. 3NP, 3-Nitropropionic acid; Cand, Candesartan

HD is significantly linked to deficits in cognitive function and exploratory behavior. Hence, the utilization of the NORT for the assessment of animals’ capacity to explore, memorize, and recognize objects (Fig. 1). Regarding the amount of time spent exploring the novel object compared to the familiar object, rats injected with 10 mg/kg 3NP displayed no significant difference. However, it significantly increased by 1.65- and 1.81-fold, with the co-treatment using 2.5 mg/kg and 5 mg/kg Candesartan, respectively. Furthermore, regarding the time spent exploring the novel object, administration of 10 mg/kg 3NP only caused the rats to spend significantly less time by 66.77%, relative to the normal group. Oppositely, it significantly elevated with the co-treatment of 2.5 mg/kg and 5 mg/kg Candesartan, by 2.22- and 2.62-fold, respectively, relative to the 3NP group. Furthermore, regarding the discrimination index, it significantly declined from 0.35 in the control group to − 0.17 in the group treated with 10 mg/kg 3NP. Conversely, co-administration of 2.5 mg/kg and 5 mg/kg Candesartan significantly improved it to 0.24 and 0.28, respectively. In addition, the total time spent exploring both objects decreased significantly by 45.56% with the 10 mg/kg 3NP treated rats compared to the positive control group. While treatment using 2.5 mg/kg and 5 mg/kg Candesartan significantly enhanced the total time spent exploring both objects by 1.46- and 1.67-fold, respectively, compared to the 3NP group. However, no statistically significant difference was observed between the two treated doses of Candesartan.

### Effect of candesartan on the open-field test in 3NP induction model

**Fig. 2:**
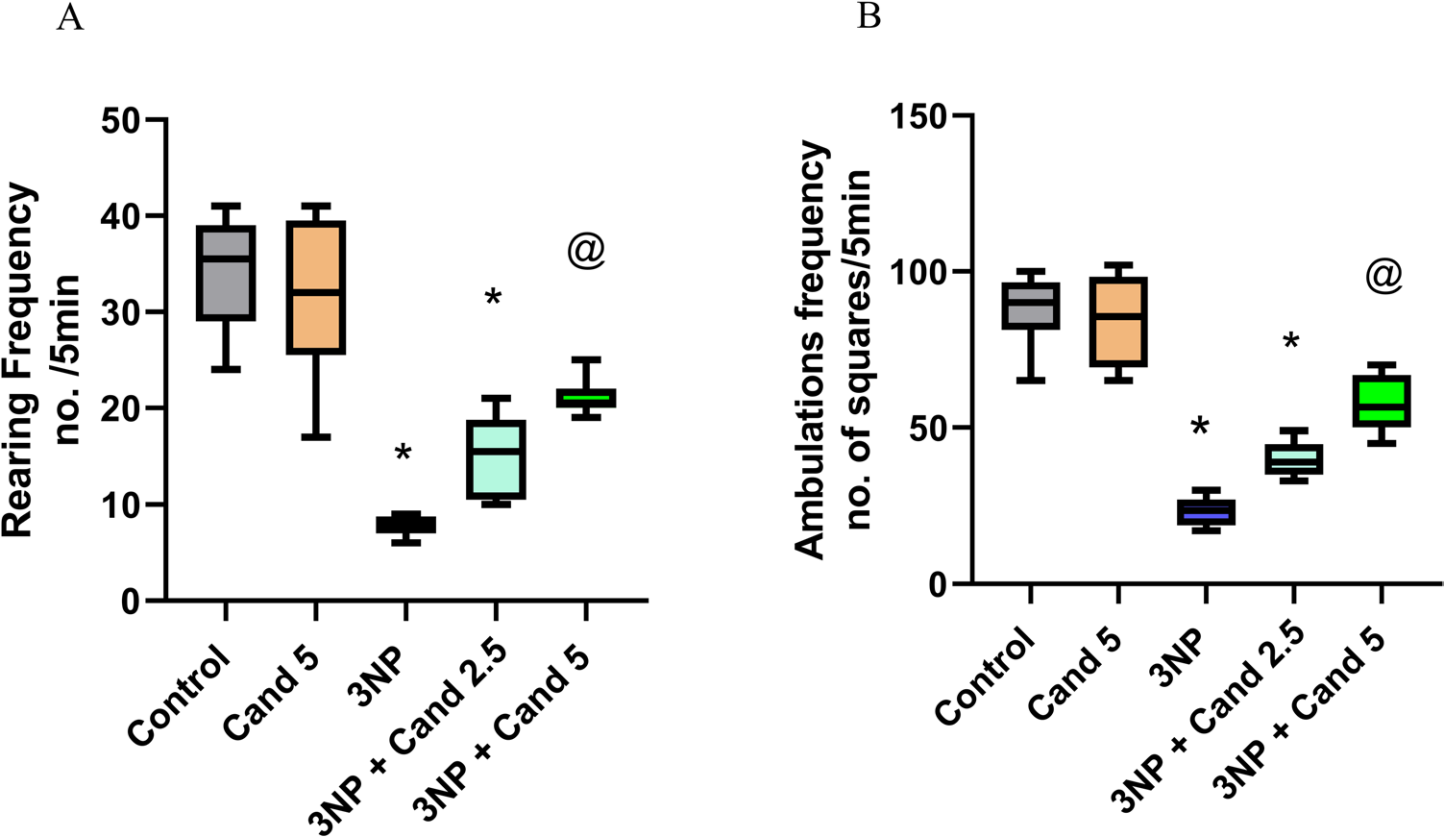
Effect of Candesartan on 3NP induced alteration in the open-field test; **A** rearing frequency, **B** ambulation’s frequency. Nonparametric data were presented as boxplots at *p* < 0.05 using the Kruskal–Wallis test followed by Dunn’s as a post hoc test. * Versus control, @ versus 3NP. 3NP, 3-Nitropropionic acid; Cand, Candesartan

One of the characteristics of HD models is locomotor and behavioral impairments, which serve as indicators of striatal atrophy and motor dysfunction, which can be displayed as gait abnormalities and hypoactivity (Fig. 2). For instance, in the open-field test, the group injected with 10 mg/kg 3NP displayed a significant decrease in the rearing frequency and ambulation frequency by 77.26% and 73.31%, respectively, compared to the normal group. However, co-administration of 5 mg/kg Candesartan led to a significant enhancement in the rearing frequency by 2.73-fold and ambulation frequency by 2.44-fold, in relation to the 3NP treated group. However, no statistically significant difference was observed between the 2.5 mg/kg Candesartan group and the diseased (3NP) group, nor between the two Candesartan-treated groups.

### Effect of Candesartan on the Morris water maze in 3NP induction model

**Fig. 3:**
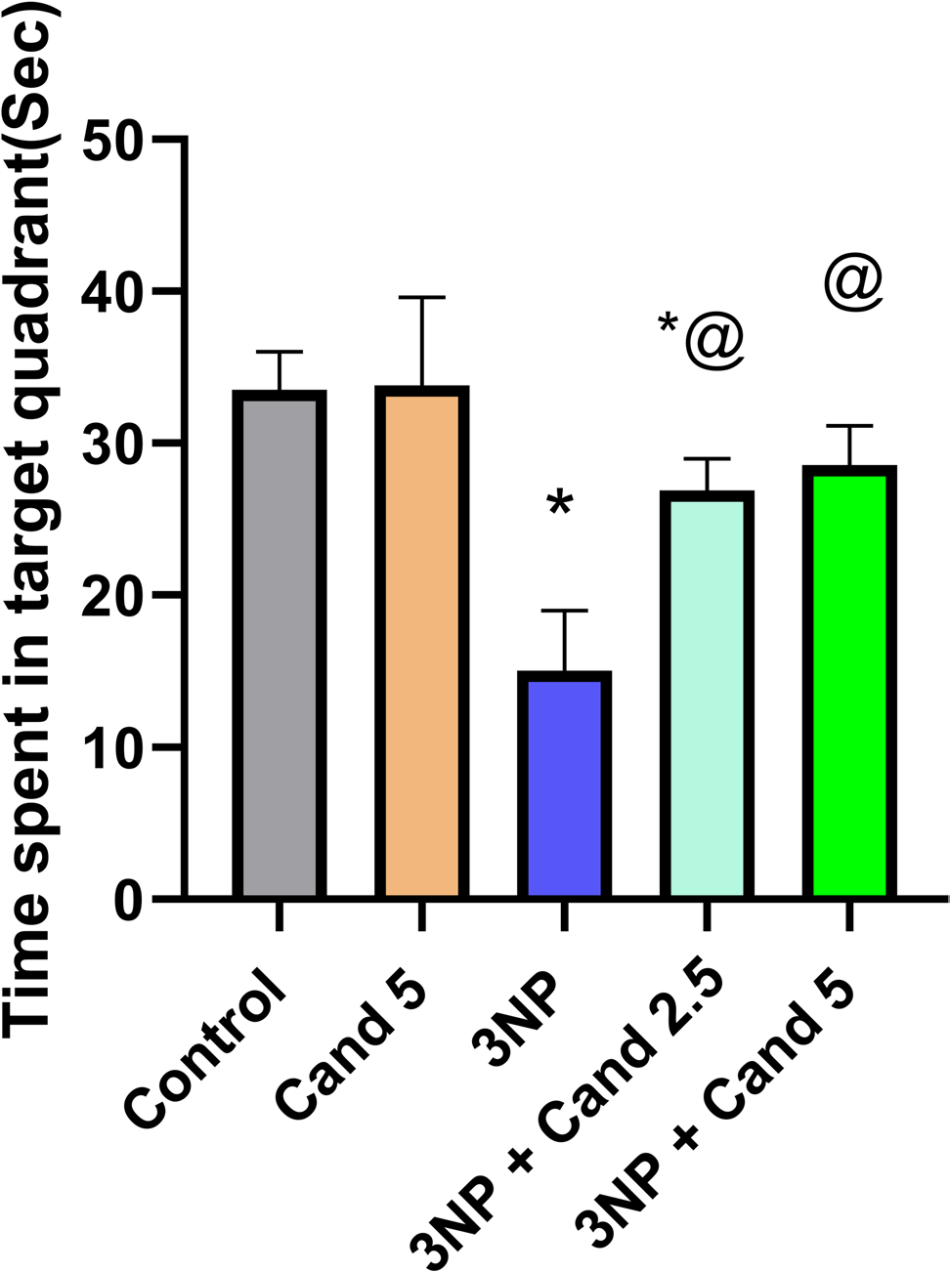
The effect of Candesartan on 3NP induced alteration in the time spent in the target quadrant in Morris water maze test. The parametric data were provided as the mean ± standard deviation at *p* < 0.05 using one-way ANOVA, followed by Tukey’s post hoc test. * Versus control, @ versus 3NP. 3NP, 3-Nitropropionic acid; Cand, Candesartan

Huntington’s disease (HD) is additionally distinguished by impaired spatial learning abilities and diminished memory function, which can be observed using Morris’s water maze test (Fig. 3). In particular, the group treated with 3NP displayed a significant decline in the time spent by the animals in the target quadrant by 55.14% as compared to the control group. Oppositely, co-administration of 2.5 mg/kg Candesartan reversed the effect of 3NP, demonstrated as a significant improvement in the time spent by the animals in the target quadrant by 1.79-fold, as related to the 3NP treated group. Moreover, co-treatment with 5 mg/kg Candesartan significantly increased the time spent by the animals in the target quadrant by 2.24-fold, in comparison to the 3NP treated group. However, no statistically significant difference was observed between the two Candesartan-treated groups.

### Effect of Candesartan on striatal histopathological examination in 3NP induction model

**Fig. 4:**
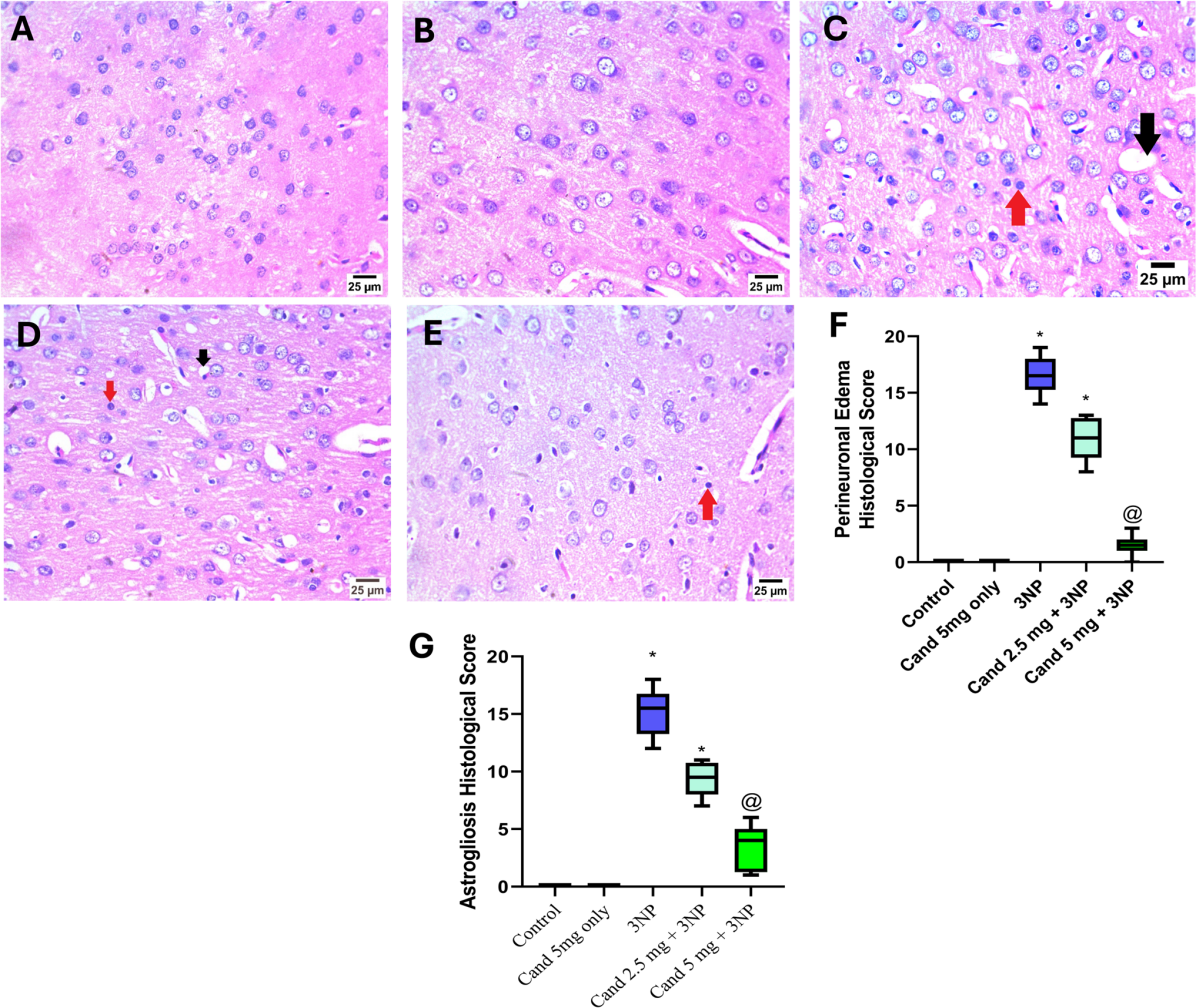
Photomicrograph showing the effect of Candesartan on 3NP induced alteration on the histological changes in the striatum with H&E (Scale bar 25 μm). **A** Control group. **B** Cand 5 group. **C** 3NP group. **D** 3NP + Cand 2.5 group. **E** 3NP + Cand 5 group. **F** Perineuronal edema histological score. **G** Astrogliosis histological score. Severe astrogliosis (red arrow) and perineuronal edema (black arrow). Nonparametric data were presented using the Kruskal–Wallis test followed by Dunn’s as a post hoc test at *p* < 0.05. * Versus control, @ versus 3NP. 3NP, 3-Nitropropionic acid; Cand, Candesartan; H&E, Hematoxylin and Eosin

Histopathological examination displayed normal histological structure in the control and group treated with Candesartan only (Fig. 4). On the contrary, the intoxication using 10 mg/kg 3NP revealed severe perineuronal edema (black arrow) and severe astrogliosis (red arrow). Interestingly, Candesartan co-treatment provoked the effect of 3NP, where administration of 2.5 mg/kg Candesartan showed moderate perineuronal edema (black arrow) which disappeared by the administration of a higher dose of 5 mg/kg Candesartan. While the severity of astrogliosis (red arrow) became moderate in the group co-treated with 2.5 mg/kg Candesartan and mild in the group co-treated with 5 mg/kg Candesartan.

### Effect of Candesartan on GFAP immunoexpression in striatum of rats in 3NP induction model

**Fig. 5:**
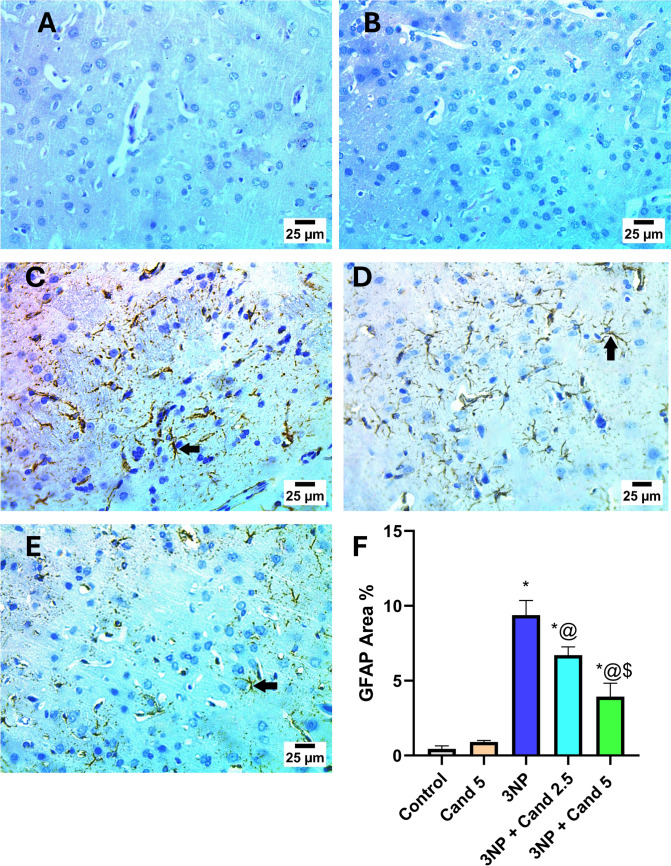
Photomicrographs represent the effect of Candesartan on 3NP induced alteration on striatal GFAP immunoreactivity. **A** Control group. **B** Cand 5 group. **C** 3NP group. **D** 3NP + Cand 2.5 group. **E** 3NP + Cand 5 group. **F** Area percent of GFAP immunoexpression. Positive reaction for GFAP in the striatum (black arrow). Data are presented as mean ± standard deviation at *p* < 0.05 using one-way ANOVA followed by Tukey’s post hoc test. * Versus control, @ versus 3NP, $ versus 3NP + Cand 2.5. 3-Nitropropionic acid; Cand, Candesartan

GFAP immunoreactivity expression in the striatum was assessed as a primary indicator of astrocyte activation, which plays a crucial role in providing functional and structural support to neurons (Fig. 5). GFAP expression was significantly augmented with the injection of 10 mg/kg 3NP by 18.32-fold, relative to the normal group. Co-treatment with 2.5 mg/kg and 5 mg/kg Candesartan significantly reduced GFAP expression by 21.31% and 50.06%, respectively, in comparison to the 3NP group. Moreover, co-administration of the higher dose 5 mg/kg Candesartan led to a significant decline in the striatal GFAP expression by 36.53% as compared to the low-dose 2.5 mg/kg Candesartan.

### Effect of Candesartan on the levels of AT2R, angiotensin-(1-7)/Mas receptor, and pS133-CREB in 3NP induction model

**Fig. 6:**
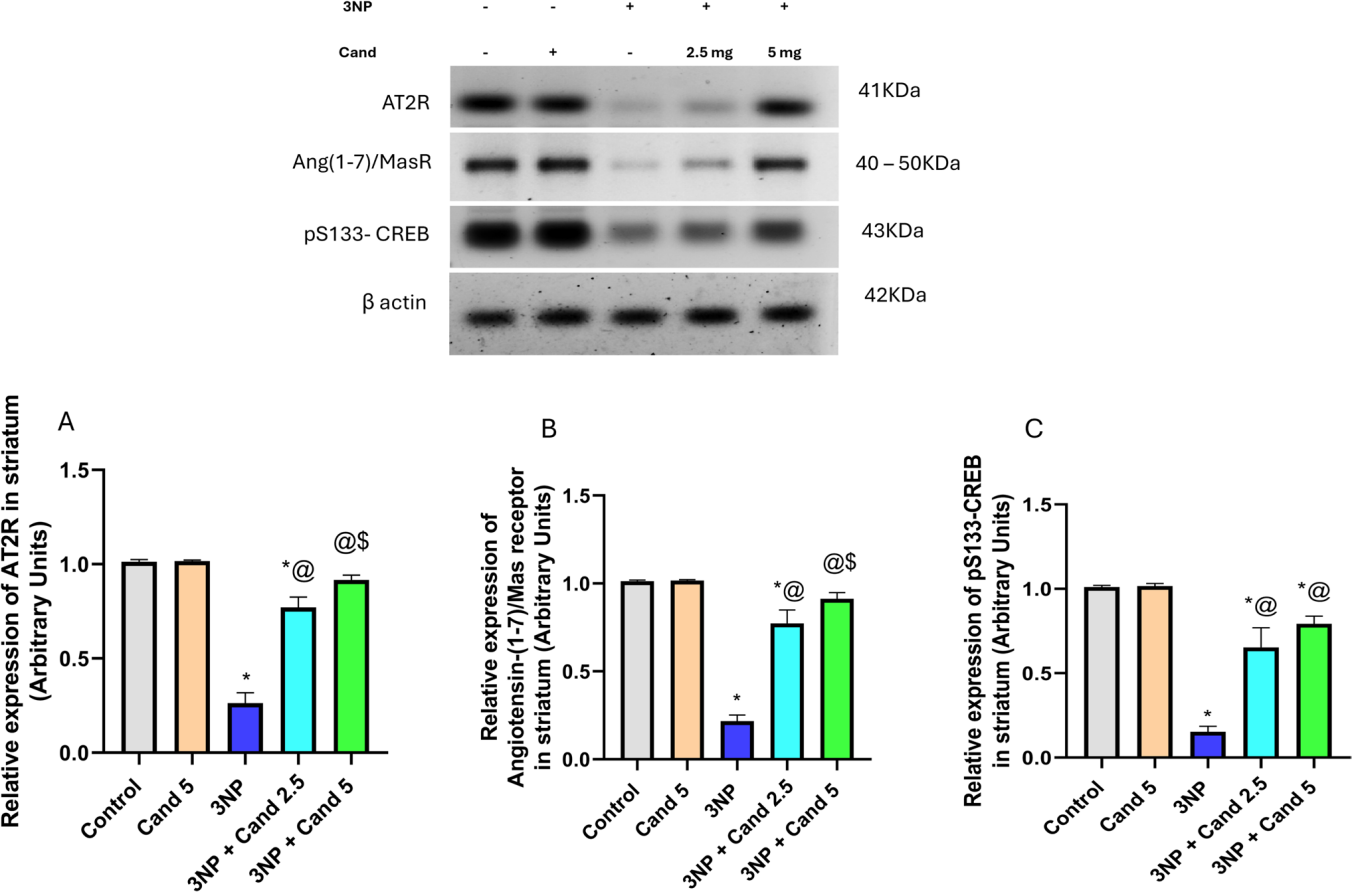
The effect of Candesartan on 3NP induced alteration on **A** AT2R, **B** Angiotensin-(1-7)/Mas receptor, and **C** CREB. The parametric data were provided as the mean ± standard deviation at *p* < 0.05 using one-way ANOVA, followed by Tukey's post hoc test. * Versus control, @ versus 3NP, $ versus 3NP + Cand 2.5. 3NP, 3-Nitropropionic acid; Cand, Candesartan; AT2R, Angiotensin II Type 2 receptor; CREB, cAMP Response Element-Binding Protein

Intoxication using 3NP caused severe neurodegeneration affecting the content of AT2R, Angiotensin-(1-7)/Mas receptor, and pS133-CREB (Fig. 6). Group injected with 10 mg/kg 3NP demonstrated a significant reduction in the content of AT2R, Angiotensin-(1-7)/Mas receptor, and pS133-CREB by 74.01%, 78.61%, and 84.81%, respectively, relative to the normal group. Oppositely, co-administration of 2.5 mg/kg Candesartan displayed a significant increase in the content of AT2R, Angiotensin-(1-7)/Mas receptor, and pS133-CREB by 2.92, 3.56, and 4.26, respectively, versus the 3NP group. Similarly, co-administration of the high-dose 5 mg/kg Candesartan illustrated a significant rise in the levels of AT2R by 3.48-fold, Angiotensin-(1-7)/Mas receptor by 4.21-fold, and pS133-CREB by 5.17-fold, relative to the 3NP group. Added to that, co-treatment with the higher dose of Candesartan (5 mg/kg) displayed a significant rise in the levels of AT2R and Angiotensin-(1-7)/Mas receptor by 1.19 and 1.18, respectively, compared to the low-dose Candesartan (2.5 mg/kg). However, no significant difference was observed in pS133-CREB expression between the two doses of Candesartan.

### Effect of Candesartan on the level of PGC-1α and BDNF in 3NP induction model

**Fig. 7:**
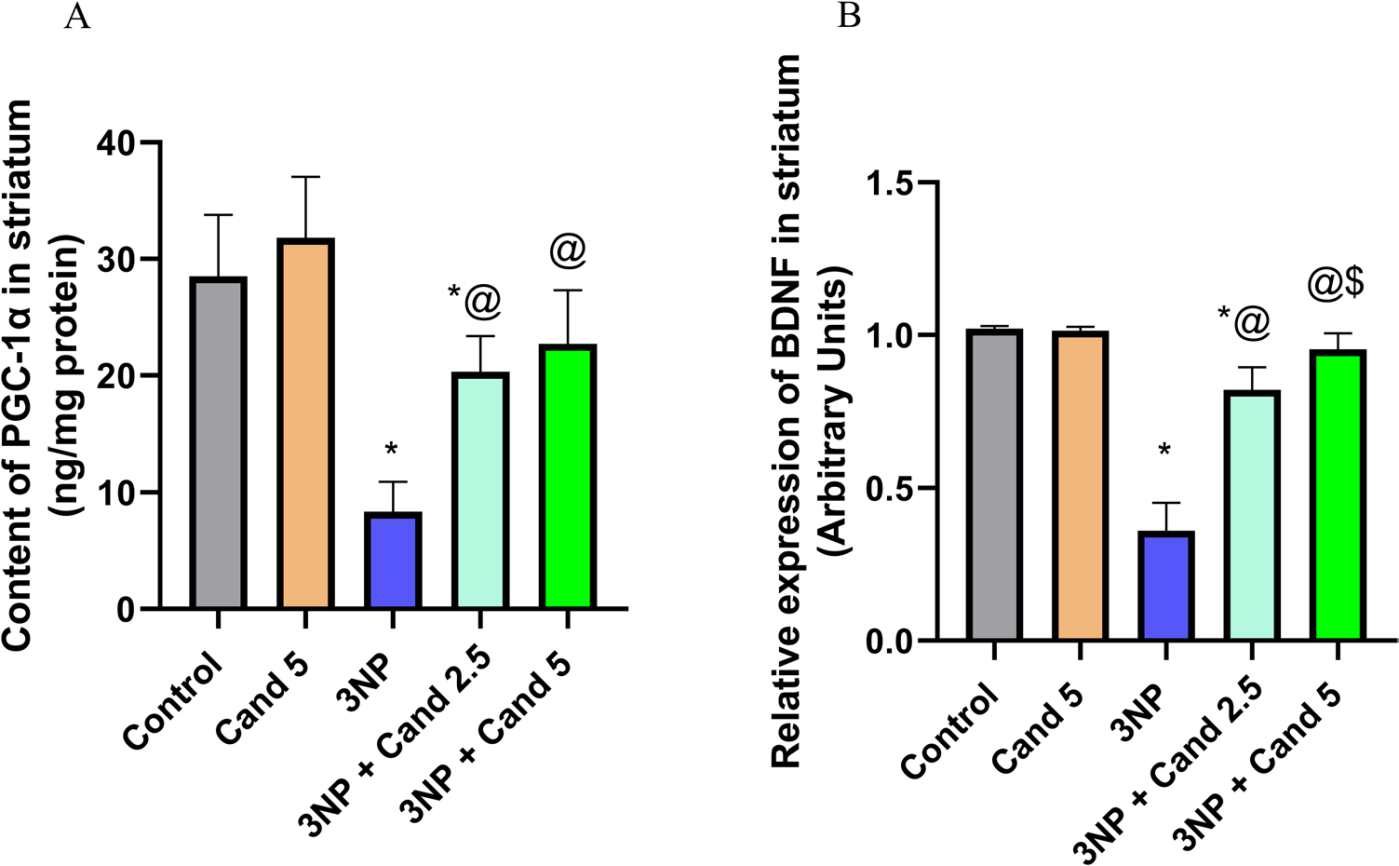
The effect of Candesartan on 3NP induced alteration on **A** PGC-1α and **B** BDNF. The parametric data were provided as the mean ± standard deviation at *p* < 0.05 using one-way ANOVA, followed by Tukey's post hoc test. * Versus control, @ versus 3NP. 3NP, 3-Nitropropionic acid; Cand, Candesartan; PGC-1α, Peroxisome proliferator-activated receptor gamma coactivator 1 alpha; BDNF, brain-derived neurotrophic factor

Intoxication using 3NP caused severe neurodegeneration as well as mitochondrial dysfunction (Fig. 7). Such effect was obvious with the injection of 10 mg/kg 3NP, which displayed a significant decline in levels of PGC-1α and BDNF by 70.76% and 64.7%, respectively, compared to the positive control group. In contrast, this decrement was abolished by the co-treatment with 2.5 mg/kg Candesartan, which demonstrated a significant increment in the content of PGC-1α and BDNF by 2.44- and 2.27-fold, respectively, relative to the 3NP group. Similarly, the co-treatment with high-dose 5 mg/kg Candesartan showed a significant elevation in the content of PGC-1α by 2.72-fold and BDNF by 2.64-fold, in comparison to the 3NP group. However, no statistically significant difference was observed in PGC-1α and BDNF between the two Candesartan-treated groups.

### Effect of Candesartan on the levels of antioxidants HO-1 and NQO1 and inflammatory mediators p65 NF-κB and IL-1β in 3NP induction model

**Fig. 8:**
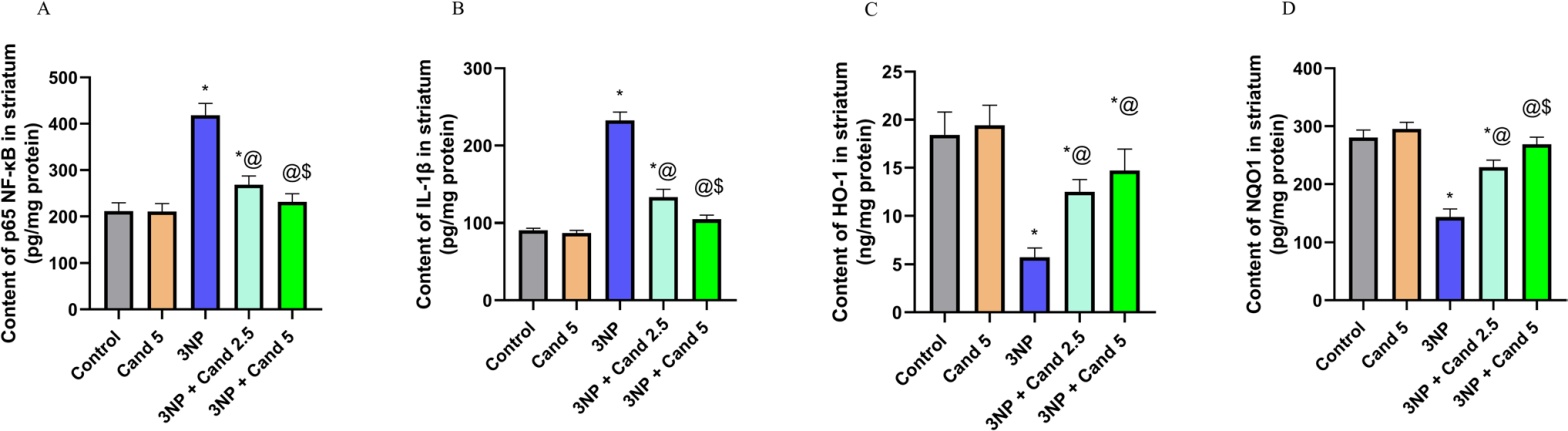
The effect of Candesartan on 3NP induced alteration on inflammatory mediators and antioxidants **A** NF-κB, **B** IL-1β, **C** HO-1, and **D** NQO1. The parametric data were provided as the mean ± standard deviation at *p* < 0.05 using one-way ANOVA, followed by Tukey's post hoc test. *Versus control, @ versus 3NP, $ versus 3NP + Cand 2.5. 3NP. 3-Nitropropionic acid; Cand, Candesartan; NF-κB, Nuclear Factor-kappa B; IL-1β, Interleukin-1 beta; HO-1, Heme Oxygenase 1; NQO1, NAD(P)H Quinone Dehydrogenase 1

The primary mechanism of toxicity associated with 3NP involves the notable impairment of the respiratory chain inside mitochondria, resulting in a reduction of striatal function with the disruption of the antioxidant activity and elevation of the inflammatory mediators (Fig. 8). This is demonstrated with a significant decline in the antioxidants levels as HO-1 and NQO1 by 68.87% and 48.96%, respectively, in the group treated with 10 mg/kg 3NP compared to the control group. Moreover, 10 mg/kg 3NP intoxication caused significant elevation in the inflammatory mediator’s level as p65 NF-κB and IL-1β by 1.98- and 2.57-fold, respectively, when compared to the control group. However, the co-treatment of Candesartan resulted in enhanced mitochondrial function, enhanced the activity of the antioxidant system, and decreased the levels of inflammatory mediators. This is displayed by a significant increase in HO-1 level by 2.17-fold and NQO1 level by 1.6-fold, accompanied with a significant decline in the levels of p65 NF-κB by 35.83% and IL-1β by 42.79% with the co-treatment of 2.5 mg/kg Candesartan compared to the 3NP group. Similarly, treatment with high-dose 5 mg/kg Candesartan showed a significant rise in HO-1 and NQO1 levels by 2.56- and 1.87-fold, respectively, with a significant decrease in the p65 NF-κB and IL-1β levels by 44.71% and 54.98%, respectively, when compared against the 3NP group. In addition, co-treatment using high Candesartan (5 mg/kg) group revealed a significant rise in NQO1 level by 1.17, associated with a significant decline in the p65 NF-κB and IL-1β levels by 13.82% and 21.31%, respectively, in comparison to the low-dose Candesartan (2.5 mg/kg). While no significant difference is observed in HO-1 expression between the two Candesartan-treated groups.

#### Effect of candesartan on the levels of JNK, c-Jun, caspase-3, and survivin in 3NP induction model

**Fig. 9:**
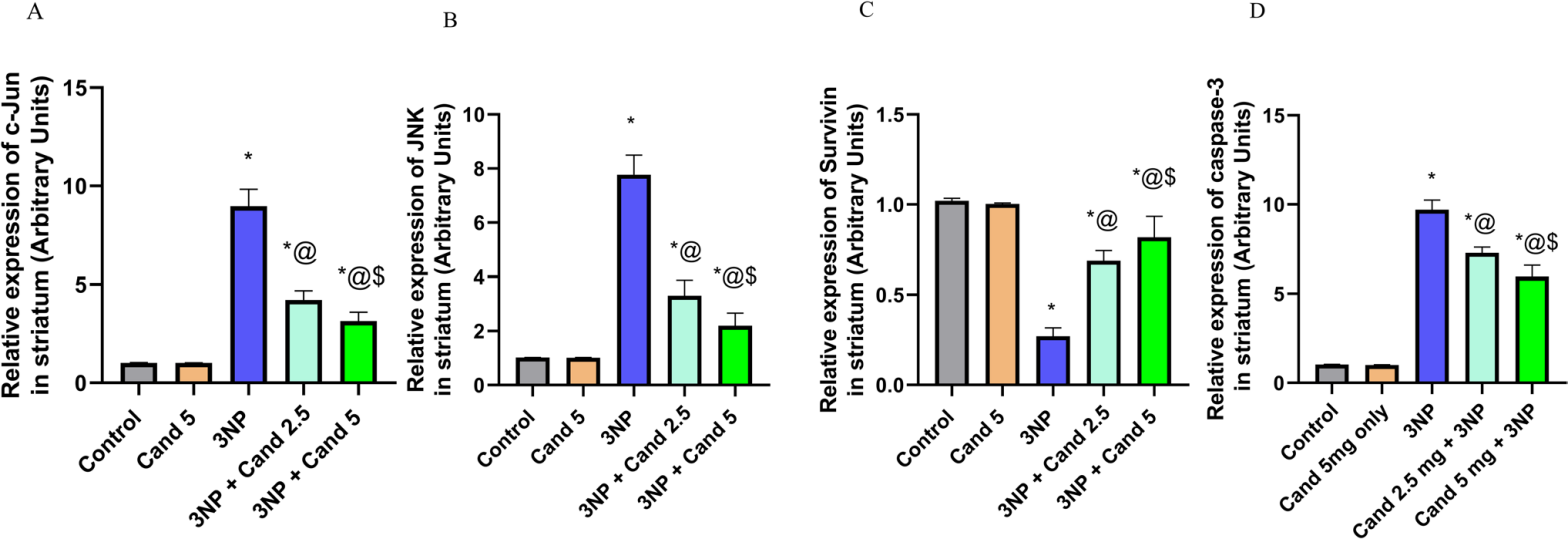
The effect of Candesartan on 3NP induced alteration in mRNA expression of **A** c-Jun, **B** JNK, **C** Survivin, and **D** caspase-3. The parametric data were provided as the mean ± standard deviation at *p* < 0.05 using one-way ANOVA, followed by Tukey’s post hoc test. * Versus control, @ versus 3NP, $ versus 3NP + Cand 2.5. 3-Nitropropionic acid; Cand, Candesartan; JNK, c-Jun N-terminal kinase

Neurodegeneration associated with HD is markedly connected to the activation of the JNK pathway, phosphorylation of c-Jun and caspase-3, as well as inhibition of survivin expression (Fig. 9). Intoxication using 10 mg/kg 3NP caused a significant increase in the expression of JNK (7.63-fold), c-Jun (8.78-fold), and caspase-3 (9.41-fold) accompanied by a significant decrease in the expression of survivin by 73.53%, in comparison to the normal group. However, co-administration of 2.5 mg/kg Candesartan abolished this effect, causing a significant reduction in the expression of JNK (57.51%), c-Jun (52.98%), and caspase-3 (25%) associated with a significant elevation in survivin (2.54 folds), in comparison to the 3NP group. Similarly, co-administration of Candesartan (5 mg/kg) illustrated a significant drop in the expression level of JNK (71.71%), c-Jun (64.96%), and caspase-3 (38.63%) together with the elevation in survivin expression (3.04-fold), compared to the 3NP group. Furthermore, co-treatment of higher dose Candesartan (5 mg/kg) displayed a significant reduction in the expression of JNK (33.43%) and caspase-3 (18.17%), versus the low-dose Candesartan (2.5 mg/kg). However, no statistically significant difference was observed in c-Jun and survivin expression levels between the 2.5 and 5 mg/kg Candesartan treatment groups.

## Discussion

The current study provides novel insights into the potential biological mechanisms behind the neurotherapeutic efficacy of Candesartan against 3NP induced neurotoxic model of HD. The results indicated that the treatment using Candesartan effectively mitigated the behavioral abnormalities and neuronal degeneration, which was verified by a multitude of incidents: (1) amelioration of cognitive and motor impairments; (2) significant improvement in the histological and immunohistopathological observations; (3) activation of the neuroprotective pathway Ang II/AT2R/Ang-(1-7)/Mas receptor and CREB/BDNF/PGC1-alpha that promote neurogenesis; (4) suppression of mitochondrial dysfunction and oxidative stress in the striatum through the restoration of HO1 and NQO1 activities, as well as inhibition of JNK/c-Jun pathway together with the inflammatory mediators.

The striatum is considered the major region of the basal ganglia that regulates motor coordination. Accordingly, experimental studies involving the administration of 3NP have induced striatal lesions, resulting in motor deficits, cognitive decline, and impaired memory retention (Jang and Cho 2016; Blum et al. 2002; D’Egidio et al. 2023). Likewise, previous studies have indicated that 3NP induces damage to the pyramidal neurons in the CA1 and CA3 regions of the hippocampus, which are known to be associated with cognitive performance (Danduga et al. 2018). The current study employed the utilization of 3NP (10 mg/kg/day for 14 days) as an induction model for HD, which consequently led to several behavioral impairments, and neurochemical and pathological alterations causing cognitive impairments and loss of memory.

While the 3NP model does not reproduce the genetic underpinnings of HD, such as the presence of mutant huntingtin aggregates, it offers a well-characterized, reproducible platform to investigate the neurotoxic cascade driven by mitochondrial dysfunction. Mitochondrial dysfunction is a central feature of HD pathogenesis, contributing to impaired energy metabolism, increased ROS production, and progressive neuronal loss. In HD patients, several enzymes in the tricarboxylic acid (TCA) cycle and the mitochondrial electron transport chain (ETC) are downregulated, resulting in reduced ATP synthesis and elevated oxidative stress. 3-NP mimics these metabolic defects by irreversibly inhibiting succinate dehydrogenase (complex II of the ETC), disrupting both the TCA cycle and oxidative phosphorylation. This leads to mitochondrial electron leakage, ROS overproduction, and energy failure, which collectively trigger neuronal apoptosis and striatal degeneration features that overlap strongly with the neurobiology of HD (Ramaswamy et al. 2007).

In the current study, the novel object recognition, open field, and Morris water maze tests were used as assessment tools to examine motor, behavioral, and cognitive impairments. 3NP administration significantly decreased the time spent exploring the novel object and the total time exploring both objects as well as the discrimination index in the NORT. Similarly, 3NP administration significantly reduced rearing and ambulation frequency and total time spent in the target quadrant in the open-field test and Morris water maze test, respectively. Interestingly, co-administration of Candesartan treatment resulted in significant enhancement in locomotor activities, memory retention, spatial learning, and cognitive function when compared to rats treated with 3NP, with major prominent effect at the highest dose (5 mg/kg). This suggests a positive impact of Candesartan treatment on these memory, cognitive, and behavioral measures, which comes in line with a previous study (Trofimiuk et al. 2018).

Moreover, histopathological assessments of the striatum in rats injected with 3NP displayed severe perineuronal edema and astrogliosis. Correspondingly, immunoreactivity analysis of GFAP expression in the striatum revealed significant expression of GFAP in the 3NP treated group. These pathological and immunohistochemical changes were ameliorated in the 3NP group treated with Candesartan (2.5 mg/kg/day and 5 mg/kg/day for 14 days). Additionally, the administration of 3NP resulted in a substantial decrease in the ultimate body weight, which is one of the features of HD (Mahdi et al. 2023). Co-treatment with Candesartan restored the body weight in comparison to diseased rats with a more noticeable impact at the greater dose (5 mg/kg).

The constituents of the RAS were implicated in various neuropsychiatric and neurodegenerative conditions, including depression and anxiety (Almeida-Santos et al. 2016; Kangussu et al. 2017), Alzheimer’s disease (Jiang et al. 2016; Tian et al. 2012), Parkinson’s disease (Rocha et al. 2016), and Huntington’s disease (Mello et al. 2017b). Moreover, previous literature has reported that the Ang II/AT2R/Ang-(1-7)/Mas receptor axis possesses neuroprotective properties (Miranda and Teixeira 2023; Gironacci, et al. 2018; Xu et al. 2011). It was previously mentioned that the AT2R activation in the brain displayed several outcomes; it reduced the generation of reactive oxygen species (ROS), promoted the survival of neuronal cells, and decreased the levels of pro-inflammatory cytokines (Shan et al. 2018; Fouda et al. 2017; Dai 2016; Khorooshi et al. 2020). Similarly, the activation of Ang-(1-7)/Mas receptors displayed specific effects on brain cells. These effects included a decrease in nuclear and mitochondrial superoxide production (Costa-Besada et al. 2018), as well as improved survival of neurons accompanied by a decrease in the level of oxidative stress in the brain (Mo et al. 2019). In addition, this signaling reduced microglial activation and astrogliosis, leading to improved neuronal density after traumatic brain injury (Janatpour et al. 2019). Interestingly, the activation of AT2R and Ang-(1-7)/Mas receptors stimulates the PI3K/Akt pathway, which subsequently stimulates the phosphorylation of CREB (Sakamoto et al. 2011; Wu et al. 2016; Mateos et al. 2016). The involvement of phosphorylated CREB is significant in the promotion of neuronal survival through the transcriptional activation of BDNF along with its receptor TrKB (Rabie et al. 2018; Song et al. 2015). It is noteworthy to emphasize that the upregulation of BDNF leads to the stimulation of neurogenesis and the initiation of TrKB phosphorylation, which serves as a positive feedback mechanism, subsequently reactivating Ang-(1-7)/Mas receptor axis, ultimately promoting the survival of neurons (Rabie et al. 2018; Yao et al. 2012). Interestingly, the administration of AT1R blockers resulted in a surge in the concentrations of Ang II; consequently, Ang II is converted to produce Ang-(1-7) which in turn is also reported to stimulate the AT2R (Hashikawa-hobara et al. 2012; Clark and Khalil 2024).

Our study revealed that co-administration of Candesartan induced the expression of Ang-(1-7)/MasR axis, as well as AT2 receptor, with the higher dose (5 mg/kg) exhibiting a significant and remarkable influence. These results were consistent with the previous studies (Widdop et al. 2002; Callera et al. 2016). Based on the previous data, our study displayed a high level of CREB and expression of BDNF with Candesartan treatment, with the higher dose succeeding to normalize CREB level and BDNF expression. These results were in harmony with the previous study (Goel et al. 2018).

PGC-1α, a transcriptional coactivator, carries a significant function in the process of mitochondrial biogenesis and the maintenance of brain energy homeostasis. Its ability to enhance mitochondrial electron transport activity and activate a comprehensive anti-ROS program renders it a highly desirable protein for regulating or mitigating the detrimental effects linked to impaired mitochondrial function, which is prominent in the early stages of neurodegenerative disorders like Parkinson’s, Alzheimer’s, and Huntington’s diseases (St-Pierre et al. 2006; Carmo 2018). In addition, the suppression of PGC-1α expression by 3NP has been reported in previous studies (Weydt et al. 2006; Ahmed et al. 2016). Hence, the diminished expression of the PGC-1α protein may potentially contribute to the manifestation of mitochondrial impairment and striatal deterioration observed in rats treated with 3NP, as reported by Chen et al. (2012). However, herein, treatment using Candesartan improved the mitochondrial function through significant elevation in the protein content of the PGC-1α with a prominent effect at the higher dose of Candesartan (5 mg/kg). The observed expression of PGC-1α is induced via CREB/PGC-1α stimulation, as mentioned before by Kang et al. (2017); Fernandez-Marcos and Auwerx 2011). In the same context, the AT1R blocker Losartan was previously proven to elevate the production of PGC-1α in an insulin resistance model (Liu et al. 2021).

Additionally, NQO1 and HO-1 were analyzed to provide further details regarding the immediate impacts of Candesartan on markers of oxidative stress. According to Calabrese et al. (2008) and Yuhan et al. (2024), it has been demonstrated that NQO1 has neuroprotective properties by mitigating oxidative damage through the conversion of highly reactive quinones into less-reactive hydroquinone. In a recent study conducted by Qi et al. (2014), the involvement of HO-1 in promoting the release of neurotrophic factors was validated in mice that suffered a stroke, in which enhanced recovery was attributed to the overexpression of HO-1 through activation of BDNF-PI3K/Akt signaling in the hippocampus. Furthermore, it has been previously stated that CREB induces the activation of NQO1 and HO-1 through the stimulation of Nrf2 (Zhou et al. 2016). In the present investigation, the administration of 3NP resulted in a noteworthy decrease in the protein levels of the antioxidants NQO-1 and HO-1, consistent with the findings reported in the prior work conducted by Yuan et al. (2020). It is interesting to note that the co-administration of Candesartan resulted in a marked increase in the concentrations of NQO-1 and HO-1, particularly at the higher dosage of (5 mg/kg). The protective effect of Candesartan treatment against striatal damage induced by oxidative stress is elucidated through the activation of the CREB/Nrf2 pathway and its downstream signaling cascade (Goel et al. 2018). These findings are in agreement with a prior study conducted by Kabel and Elkhoely (2017) and Nioi et al. (2003).

The 3NP compound resembles the pathogenesis of Huntington’s disease (HD) by causing the degeneration of GABAergic neurons in the striatum. This degeneration is facilitated by the activation of microglial cells, which leads to the excessive production of cytotoxic agents including free radicals and pro-inflammatory cytokines, such as NF-κB and IL-1β (Bonsi et al. 2006; Ahuja et al. 2008; Napolitano et al. 2008). Moreover, NF-κB has a crucial role as a transcription factor in M1 macrophages, being essential for the activation of numerous inflammatory genes, particularly those responsible for encoding IL-1β (Wang et al. 2014).

In this study, 3NP intoxication caused marked elevation in the levels of NF-κB and IL-1β, which is in line with the previous study (Mustafa, et al. 2021; Benicky et al. 2011). However, co-treatment using Candesartan leads to significant inhibition in NF-κB and IL-1β with a prominent effect at a higher dose (5 mg/kg), which is consistent with previous studies (Benicky et al. 2011; Qie et al. 2020). Such a decline in the levels of NF-κB and IL-1β can be attributed to the activation of CREB, which was in line according to these studies (Dong et al. 2018; Brautigam et al. 2005; Wen et al. 2010).

The involvement of the JNK pathway has been indicated in both in vitro and in vivo experimental models of neuronal cell death in chronic neurodegenerative conditions, including Alzheimer’s disease (AD) and Parkinson’s disease (PD) (de los Reyes Corrales et al. 2021). Moreover, studies conducted on Huntington’s disease (HD) have demonstrated that the mutant huntingtin protein (htt) activates the JNK pathway in cellular models (Yan et al. 2024). The transcription factor c-Jun is the key component of the JNK pathway, and its regulation occurs at both the transcriptional and post-transcriptional stages through the activation of JNK (Davis 2000; Sun et al. 2024). Various internal and extracellular events may contribute to the activation of JNK in neuronal cells. One of these events is the generation of reactive oxygen species by 3NP, which has displayed the occurrence of nuclear translocation of activated JNK and phosphorylation of c-Jun (Perrin et al. 2009; Garcia et al. 2002). Furthermore, a previous study demonstrated that JNK/p-c-Jun activation leads to caspase-3 activation, thereby mediating apoptotic cell death. For example, JNK-driven cytochrome c release activates caspase-3 in neuronal models (Chen, et al. 2021). In the present study, intoxication with 3NP displayed significant increases in the JNK/c-Jun/caspase-3 expression, which is in line with the previous study (Garcia et al. 2002; Verdaguer et al. 2023; Elbaz et al. 2025). However, co-treatment with Candesartan mediated inhibition of the protein expression of JNK/c-Jun/caspase-3 with a prominent effect at a higher dose (5 mg/kg), which is supported by a previous study (Elkahloun et al. 2016; Ahmed and Mohamed 2019). Therefore, the mechanistic pathway behind the inhibition of JNK/c-Jun can be regarded as due to the activation of CREB as mentioned by Zhang et al. 2008a.

Survivin, a recently discovered member of the inhibitor of apoptosis protein (IAP) family, has been implicated in various neurodegenerative diseases such as Amyotrophic Lateral Sclerosis (ALS) and brain stroke (Tolosa et al. 2008; Zhang et al. 2008b). It has been suggested that reinforcing the signaling pathway of survivin could potentially contribute to the therapeutic management of neurodegenerative disorders and cognitive impairments (Chu et al. 2017). According to Baratchi et al. (2010), prior research has demonstrated that survivin functions to inhibit the activation of Caspase-3 and Caspase-7. For instance, previous studies have demonstrated that survivin exhibits neuroprotective properties in the hippocampus of a mouse model of brain stroke (Sehara et al. 2013). Additionally, it has been observed that survivin enhances antioxidant activity and reduces apoptosis, as reported in studies conducted by Baratchi et al. (2010); Baratchi et al. 2011). In a study conducted by Syahrani et al. (2020), it was observed that the suppression of survivin resulted in an elevation in oxidative stress levels and a reduction in the expression of the superoxide dismutase (SOD) gene. In the present study, the expression of survivin was significantly reduced with the administration of 3NP. However, co-treatment with Candesartan ameliorated the effect of 3NP, leading to a marked elevation in survivin expression with a prominent effect at higher dose (5 mg/kg). Such an increase can be regarded as the stimulation of PI3K/Akt by Candesartan, which causes upregulation of survivin leading to a cytoprotective effect achieved by the transcriptional activation of genes linked with antioxidants or cell survival within the nucleus; this is in line with the previous studies by Zhang et al. (2016) and Hashikawa-hobara et al. (2012).

Moreover, various RAS-targeting strategies, such as ACE inhibitors, AT₂R agonists, and MasR agonists, have demonstrated neuroprotective roles in different models of neurodegeneration. However, AT1 receptor blockade via candesartan provides distinct advantages. Unlike ACE inhibitors, which may reduce both harmful Ang II and protective Ang-(1-7), Candesartan selectively inhibits the deleterious AT1 receptor-mediated effects while preserving or enhancing Ang II availability for AT2 receptor activation and Ang-(1-7) synthesis. This allows for indirect stimulation of the protective RAS arms without reducing overall RAS tone. While direct AT2 receptor or Mas receptor agonists have shown benefit in preclinical studies, they remain clinically unavailable (Rodríguez-Pallares et al. 2025).

In conclusion, the results of the present investigation offer the initial and first empirical support for the neuroprotective impact of Candesartan in mitigating striatal degeneration and mitochondrial dysfunction induced by 3NP, hence suppressing the progression of Huntington’s disease through the activation of AngII/AT2R/Ang-(1-7)/MasR/CREB/BDNF pathway as well as involvement of JNK/c-Jun and survivin, offering new prospective and possibility for the use of AT1 receptor blockers in the treatment of HD.

## Study limitations and future implications

One limitation of the present study is the lack of direct mechanistic confirmation regarding the effect of candesartan on survivin expression. While survivin is a key anti-apoptotic marker, its regulation by candesartan remains speculative and warrants further investigation, especially considering that most studies on survivin have focused on oncology rather than neurodegeneration.

Additionally, clinical translation requires evaluation of Candesartan’s effects in HD patients with comorbid hypertension, since blood pressure modulation could influence both CNS and cardiovascular outcomes. This is particularly relevant given that mutant huntingtin is expressed in cardiomyocytes, contributing to cardiac dysfunction and hypertension, which are among the leading causes of mortality in HD patients. Moreover, cardiovascular diseases (CVD) can accelerate neurodegenerative progression by impairing cerebral perfusion and promoting systemic inflammation.

Therefore, future studies should explore the dual neuroprotective and cardioprotective potential of candesartan in translational HD models and in clinical settings where neurodegeneration and cardiovascular dysfunction intersect. Investigating candesartan’s role in both central and peripheral RAS modulation may open new therapeutic avenues for HD patients, particularly those at risk for or suffering from cardiovascular complications.

## Supplementary Information

Below is the link to the electronic supplementary material.

Supplementary file1 (DOCX 4678 kb)

## Data Availability

Data can be made availabe on request.
