# Supplementary material for: Neuroprotective effects of candesartan in 3-nitropropionic acid-induced Huntington’s disease: modulation of angiotensin and CREB/BDNF/PGC1-α signaling
Source: Inflammopharmacology. 2025 Sep 16;33(10):6231–49. doi: 10.1007/s10787-025-01889-6 (PMC12552329; doi:10.1007/s10787-025-01889-6)
Supplement: Supplementary file 1 — Supplementary file1 (DOCX 4678 kb) [file 10787_2025_1889_MOESM1_ESM.docx]

**Uncropped western blot gels**


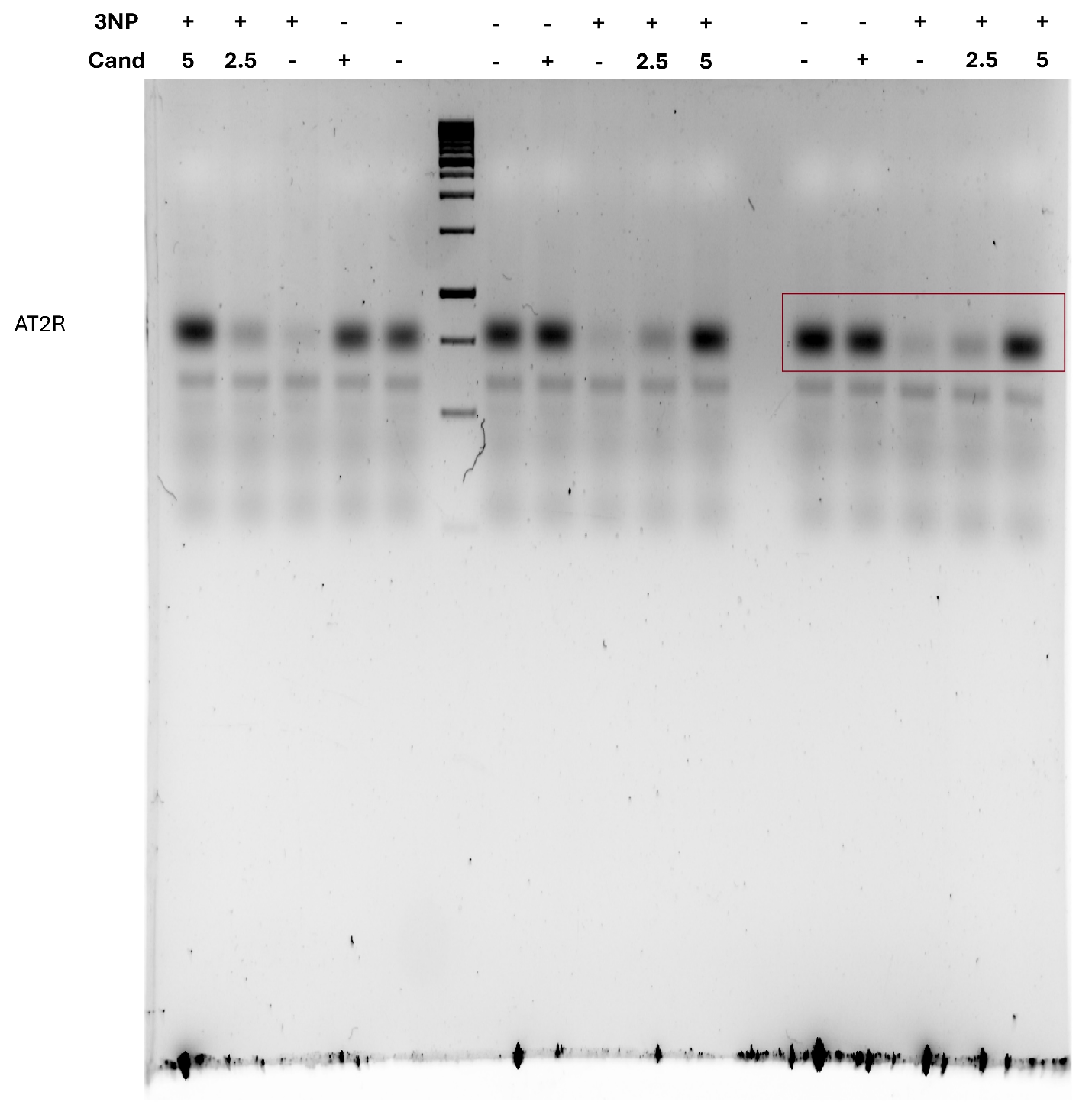


Figure 1 uncropped gel of AT2R


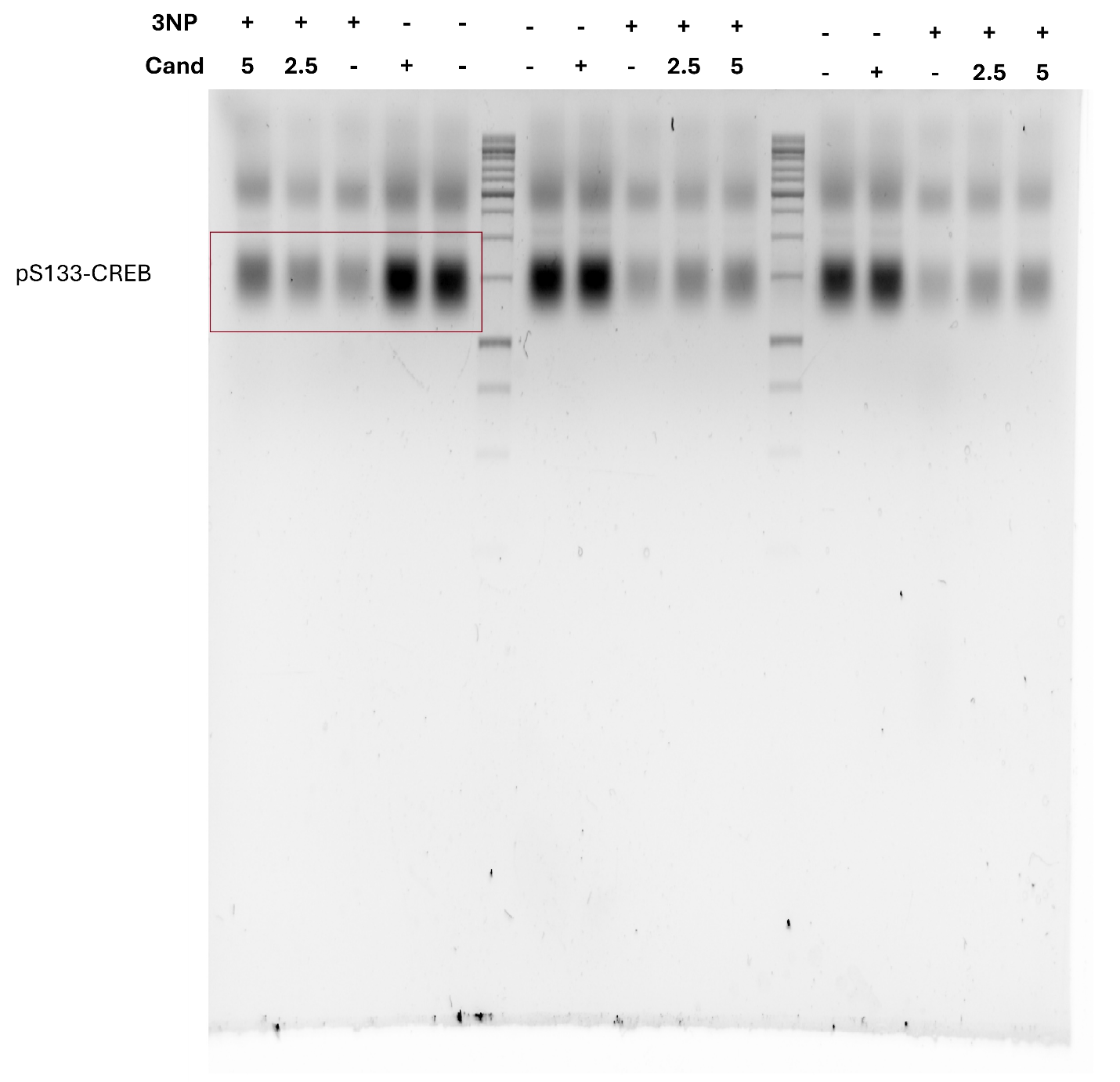


Figure 2 uncropped gel of pS133-CREB


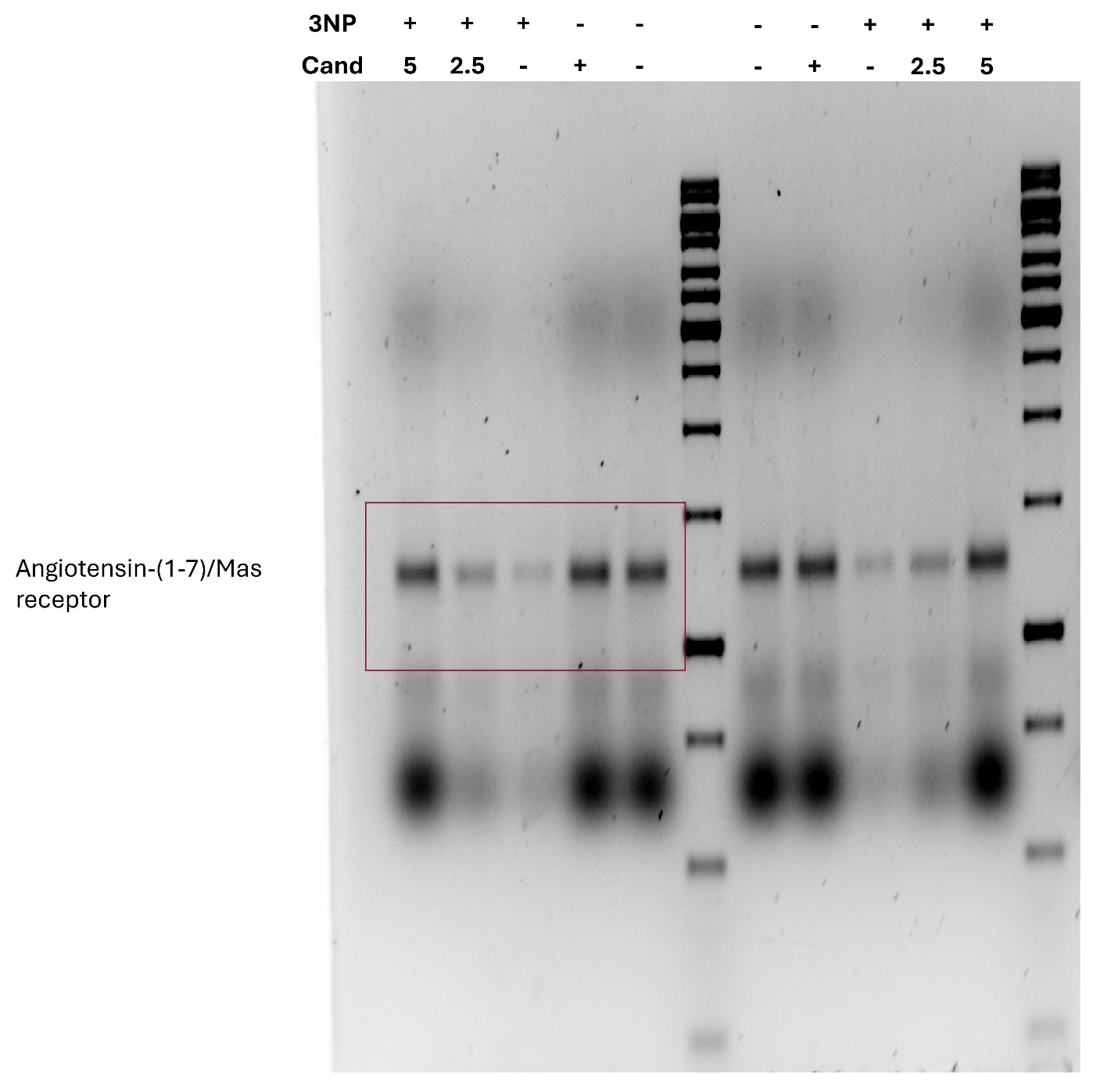


Figure 3 uncropped gel of Angiotensin-(1-7)/Mas receptor


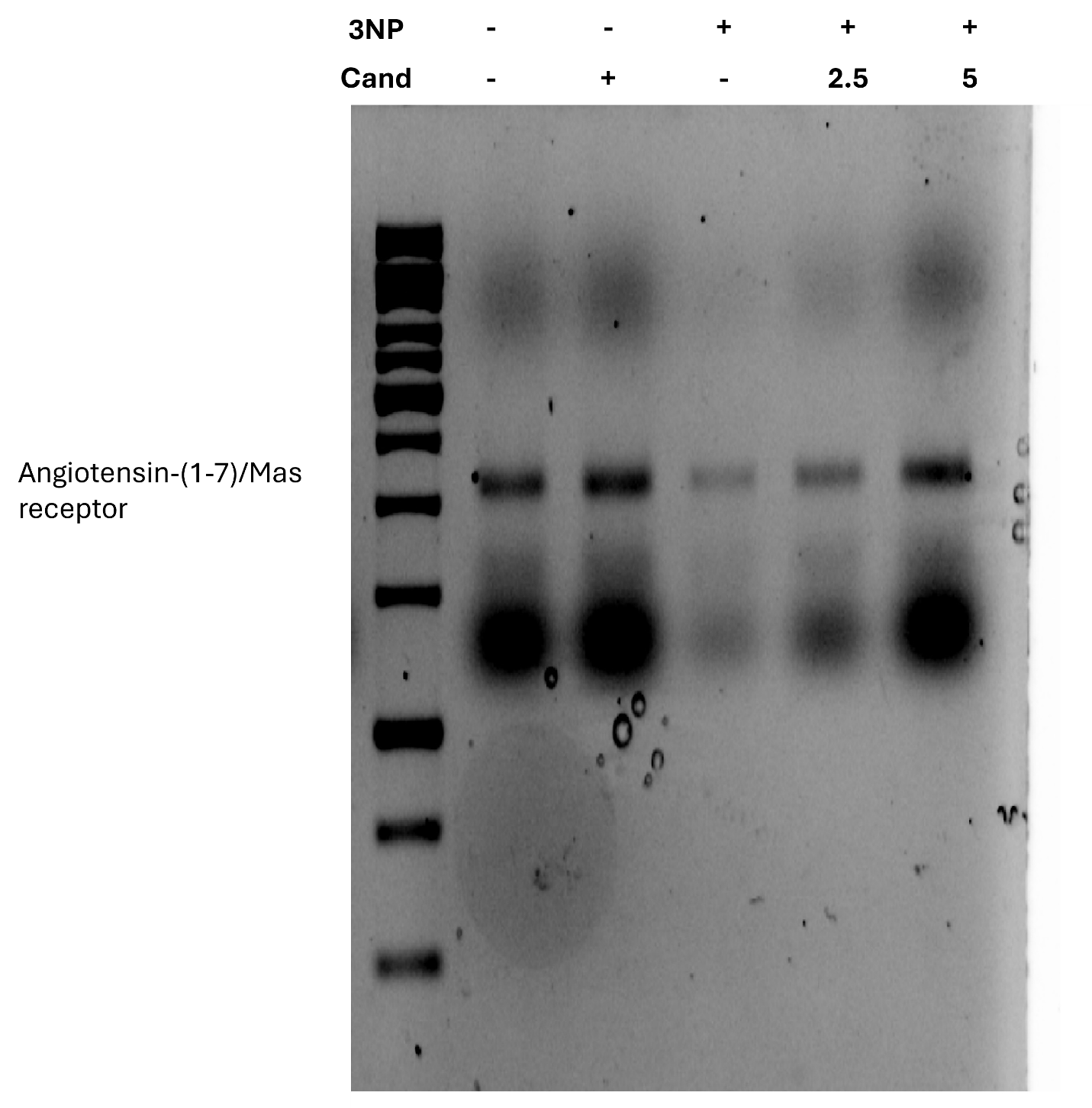


Figure 4 uncropped gel of Angiotensin-(1-7)/Mas receptor


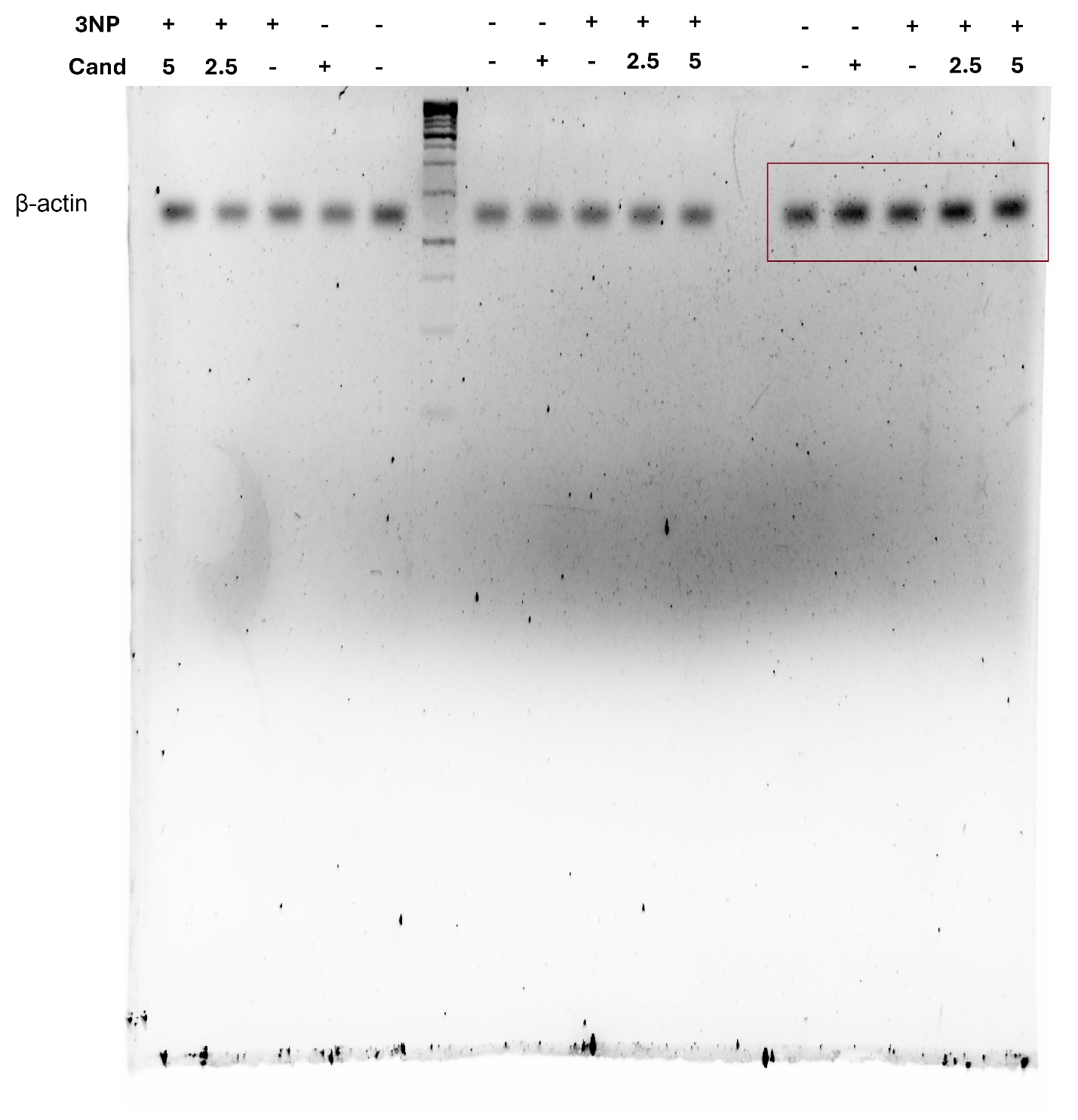


Figure 5 uncropped gel of β-actin
